# How Outpatient Palliative Care Teleconsultation Facilitates Empathic Patient-Professional Relationships: A Qualitative Study

**DOI:** 10.1371/journal.pone.0124387

**Published:** 2015-04-22

**Authors:** Jelle van Gurp, Martine van Selm, Kris Vissers, Evert van Leeuwen, Jeroen Hasselaar

**Affiliations:** 1 Department of Anesthesiology, Pain, and Palliative Medicine, Radboud University Medical Center, Nijmegen, Netherlands; 2 Amsterdam School of Communication Research, University of Amsterdam, Amsterdam, Netherlands; 3 Department of IQ Healthcare Ethics Section, Radboud University Medical Center, Nijmegen, Netherlands; The University of Queensland, AUSTRALIA

## Abstract

**Objective:**

The problems and needs of advanced cancer patients and proxies normally increase as the disease progresses. Home-based advanced cancer patients and their proxies benefit from collaborations between primary care physicians and hospital-based palliative care specialists when confronted with complex problems in the last phase of life. Telemedicine might facilitate direct, patient-centered communication between patients and proxies, primary care physicians, and specialist palliative care teams (SPCTs). This study focuses on the impact of teleconsultation technologies on the relationships between home-based palliative care patients and hospital-based palliative care specialists.

**Methods:**

This work consists of a qualitative study among patients, family members, and caregivers that utilizes long-term direct observations, semi-structured interviews, and open interviews following the observations.

**Results:**

The analysis of the empirical data resulted in three key concepts that describe the impact of teleconsultation on the patient-professional relationship in palliative homecare: *transcending the institutional walls of home and hospital; transparency of teleconsultation technology*; and *technologized*, *intimate patient-professional relationships*. Teleconsultation offers (1) condensed encounters between home-based palliative care patients and distant professionals, (2) a unique insight into the patients’ daily lives for palliative care specialists, and (3) long-term interaction that results in trustful relationships and experiences of intimacy and relief.

**Conclusions:**

Teleconsultation fits the practice of home-based palliative care. Teleconsultation can, if well applied, facilitate computer-mediated but empathic patient-palliative care specialist relationships, which enable professional care attuned to the patient’s context as well as patient involvement. This article proposes a teleconsultation implementation guide for optimal use of teleconsultation in daily palliative care practice.

## Background

Patients with advanced cancer mostly suffer from severe physical and psychosocial problems as the disease progresses [1]. These problems require a palliative care approach that strongly focuses on the maintenance of patients’ and proxies’ quality of life [2]. Home-based advanced cancer patients usually rely on their primary care physicians for community-based palliative care. Specialist palliative care is not always easily and/or timely accessible to them because a part of the primary care physicians ignore collaborative care networks [3,4] or functional decline hampers patient visits to outpatient palliative care facilities. However, specialist care is desirable when complex problems arise [5,6]. Therefore, experts in the field emphasize collaboration between patients, primary care physicians, and specialist palliative care services to guarantee continuous high-quality care in the community [5,6–8]. How home-based palliative care patients can present themselves within such collaborations remains unclear, especially as these vulnerable and often immobile patients usually cannot travel to multidisciplinary care settings. In this regard, telemedicine may be promising for the provision of multidisciplinary palliative care, as it enables patient involvement at a distance.

Telemedicine technologies are known to facilitate the support for and monitoring of home-based palliative care patients. For example, online monitoring enhances patients’ incorporation of illness(es) into their daily routines and yields improvements in cancer-related pain and depression [9,10]. Furthermore, a “telehospice” project found that patients and caregivers were enthusiastic about the “instant face-to-face communication” with a hospice worker via the use of videophone technology [11]. A qualitative study of chronic obstructive pulmonary disease (COPD) showed that the use of webcams for patient-professional contact contributed to concentrated conversations, revealed non-verbal cues and opened up personal spaces [12]. However, telemedicine’s potential to intensify caring relationships is accompanied by the fear that technologized services dehumanize patient-professional relationships [13–17] by imposing protocols that obstruct compassionate palliative care [18–20] and disrupting patients’ home lives [11]. These fears have hindered the widespread implementation of telemedicine in palliative care settings [21,22]. Hence, this study examined how telemedicine facilitates or limits the patient-professional relationship, which is considered the key to palliative care [20]. In particular, this study investigates the practical and normative fit of weekly real-time audiovisual teleconsultations between home-based patients who require palliative care, their primary care physicians, and hospital-based palliative care specialists [16].

## Methods

This study’s qualitative design included long-term observations of ‘home-based patient-specialist palliative care team (SPCT) clinician’ communication during teleconsultations and serial semi-structured interviews with patients, family caregivers, primary care physicians (PCP), and SPCT clinicians. The Medical Research Ethics Committee of Arnhem-Nijmegen, Netherlands, approved this study and the consent procedure described below (NL32164.091.10) in September 2010. The patients and informal caregivers were included after a) verbal consent from a patient’s PCP and b) written informed consent of both patient and one of his/her close informal caregivers. Data were recorded from November 2010 to March 2013.

### Teleconsultation device

**Fig 1:**
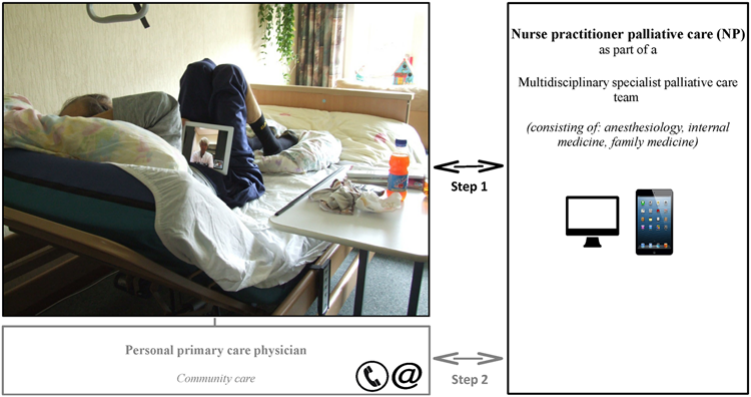
Distant care for the dying: a teleconsultation service between a specialist palliative care team, patients, family caregivers and primary care physicians. Legend: Step 1. a. The NP initiates digital bedside consultations with the patient on a regular basis (starting with 1 teleconsultation a week). b. Duration: approximately 30 minutes. c. Standardized inventory of patient's symptoms and other multidimensional problems. d. The NP provides practical advice on caring and nursing; abstains from direct medical treatment advices and decisions. *Step 2 (not the focus of this particular study)*. a. The NP discusses her findings with palliative care specialist and reports to the primary care physician. b. Involved health care professionals compose and/or discuss the treatment plan. c. As long as the patient resides at home,the primary care physician is responsible for discussing the treatment plan with the patient and together they decide about further treatment and care. **Important notes**: a. A patient cannot directly contact the SPCT via the teleconsultation route as to secure the primary care physician's central position and to prevent an overload of the care system. b. In case the primary care physician participated 'real time' by visiting the patient at home during teleconsultations, the teleconsultation with a patient/consultation with a primary care physician/feedback to the patient was compressed into a single interaction.

Weekly videoconferencing interactions were performed between a hospital-based SPCT and palliative care patients living at home (Fig 1). PCPs were invited to attend the teleconsultations at the patient’s home. If PCPs could not be present online, the SPCT subsequently shared the content of these teleconsultations with the PCPs. Due to rapid technological developments, two teleconsultation devices were used during the course of this study: 1) a simplified desktop computer called a “Bidibox” (Focuscura Inc.; Netherlands) and 2) an iPad 2 (Apple Inc.; United States). The change to a tablet computer was inspired by the clear advantages of tablet computers for palliative care patients.

The teleconsultation service was implemented in the daily Dutch palliative care practice, in which primary care physicians have full responsibility for *home-based* patients (including regular contacts and home visits). Primary care physicians can use telephone consultations with more specialized colleagues for additional palliative care expertise. If a home-based palliative care patient is discharged from a hospital with a specialist palliative care team, he/she might still occasionally visit this hospital-based team as an outpatient or the team might stay in touch with the patient by phone. Hospital-based palliative care team clinicians will seldom visit patients at home. If outpatients continue to contact a hospital-based specialist palliative care team, the team members and the primary care physicians should share their information to prevent parallel care.

### Patient recruitment

Home-based palliative care patients were recruited following a purposeful sampling procedure [23]. The following inclusion criteria were applied: patients a) were suffering from any advanced-stage cancer, b) had an estimated life expectancy ≤ 3 months c) had a poor functional status (Karnofsky score ≤ 60) [24] (designated in the US as “hospice appropriate”), d) lived at home under the direct supervision of their primary care physician and were supported by an informal caregiver, e) were over the age of 18, and f) were Dutch-speaking. Cognitively impaired patients were excluded from this study. In addition, two non-oncological patients with end-stage COPD (GOLD Grade 4 [25], increasing exacerbation frequency, occurrence of co-morbidity, dyspnea, and cachectic syndrome) were also recruited [26]. The death or dropout of patients defined the end of the study.

### Data collection—observations and in-depth interviews

The practice of teleconsultation was repeatedly assessed from different participant perspectives at different moments in the terminal care trajectory. During the research period, interim analyses determined the focus for further investigations. Triangulation was applied between cases (a case consisted of one patient, his/her informal care, his/her primary care physician(s), and the SPCT clinicians involved), between observational and interview data within a case, within interviews by approaching teleconsultation from different perspectives, and between the participants’ different (professional) perspectives [27–29].

Three techniques were used in this study.

#### 1. Long-term direct observations

the main researcher (JG) observed the weekly teleconsultations at the patients’ homes or alongside the SPCT clinicians. As a result, the main researcher (JG) developed long-term relationships with both the patients and involved professionals. Observations were made using an observation guide (S1 Appendix [30]). The observation guide structured the field notes, which also contained verbatim passages of the audio-recorded teleconsultations and/or open follow-up interviews.

#### 2. Semi-structured interviews

In addition to the observations, semi-structured interviews were scheduled with respondents: patients, informal caregivers, primary care physicians, and SPCT members [31,32]. With respect to patients and SPCT members, the data collection followed an iterative process: the results of earlier observations determined the number of interviews and the selection of topics from the interview guide (S2 Appendix). With respect to primary care physicians and informal caregivers, a baseline interview and an exit interview after the end of patient participation were administered.

Interviews were audio-recorded and transcribed verbatim. All data were uploaded in CAQDAS ATLAS.ti (version 6) for analysis.

#### 3. Open follow-up interviews

the researcher’s presence at a patient’s home or in the hospital before, during, and after the teleconsultations provided opportunities for open follow-up interviews regarding the participants’ experiences with the teleconsultation. The questions asked were based on to the topics from the interview guide (S2 Appendix).

### Data analysis

The qualitative data were analyzed using the open, axial, and selective coding that is common to a grounded theory approach [23,27]. The main author (JG) started with open coding of the field notes and interview transcripts from the first 12 cases. Second, a “constant comparative analysis” [27] was undertaken by JG to compare codes and accompanying quotations to categorize the codes by themes (in graphic network views in ATLAS.ti). Third, JG axially coded the data to create a coherent picture of the emerged themes, distinguishing themes from subthemes and explaining their mutual relationships. This process resulted in graphic tree structures of themes accompanied by explanatory memos, all of which were extensively discussed with the other authors. Four key concepts were constructed at the top of the tree structures. Finally, the tree structures were transformed into a classification scheme that included the four key concepts, themes, and subthemes. Members of the multidisciplinary research group then tested and valued the classification scheme. They independently coded a cross-section of the research data according to the scheme and jointly discussed their codings. Subsequently, JG utilized the classification scheme to selectively code the data from the remaining 6 cases to refine the classification scheme and test its coherence [23]. A member check was performed for the four main themes and subsequent subthemes.

## Results

A total of 18 palliative care outpatients, 16 with cancer and 2 with COPD, were included in the study. The patients’ mean age was 61 years old (range 24–85). The median time of participation was 43 days (range 7–418). Additionally, 17 informal caregivers, 15 PCPs (all related to the included patients) and 12 SPCT clinicians participated in this study (Table 1). The 18 cases produced 55 interviews, 40 open follow-up interviews, and 129 field notes

**Table 1:** Characteristics of the study participants.

*Patients* ^a^	
Number of participants	
Sex (male/female)	10/8
Age groups (number of patients; range)	
18–44 years old	3
45–64 years old	7
65+ years old	8
Informal care support	
Living with a patient	11
Family caregiver	4
Informal caregiver other than family caregiver	2
No informal care	1
Diagnosis—cancer	16
Sarcoma (osteosarcoma)	1
Gastric intestinal cancer (appendix, 2; gastric, 1; colon, 1)	4
Brain tumor (1)	1
Urogenital cancer (bladder, 1; cervix, 1; prostate, 2)	4
Head and neck cancer	2
Melanoma	1
Breast cancer	1
Pancreatic carcinoma	2
Diagnosis—COPD	2
Number of patients via recruitment strategy	
Primary care physicians	3
Palliative care nurses from renowned homecare institution	1
Specialist palliative care team	12
Specialist nurses for respiratory diseases	2
*Primary care physicians*	
Number of participants	
Independent primary care physicians	18
*SPCT members*	
Number of participants	
Palliative care physicians	8
Nurse practitioners/nurses	4

^a^Four patients far exceeded the 3-month life expectancy (range 192–418 days in the study). Twelve patients remained in the study less than 2 months; of these 12 patients, 1 left the study because of dissatisfaction, 2 left because of transfer to a hospice, and 1 left because of the prospect of receiving euthanasia.

The overall study on teleconsultation in palliative homecare produced four key concepts. Of these four key concepts, three explained ‘home-based patient-palliative care specialist’ relationships that arise from teleconsultation and were elaborated on in this paper. The three key concepts that emerged from the empirical data were: (a) *transcendence*, (b) *transparency*, and (c) *technologized*, *intimate caring relationships* (Table 2). The fourth key concept, which is about interprofessional collaboration in home-based palliative care via teleconsultation, was discussed elsewhere (unpublished data).

**Table 2:** Taxonomy of key components defining the fit of teleconsultation technology and service into the practice of palliative homecare.

*Key components*	*Themes*
**1. Transcending the institutional walls of home and hospital**	a. Surplus value of teleconsultation technology in enabling specialist care at home
	b. Teleconsultation technology’s incentive to join in condensed digital encounters
	c. The potential of teleconsultation technology to jeopardize privacy
**2. Transparency of technology defines quality of care**	a. The requirement of instant use
	b. Teleconsultation’s fit into the patients’ domestic lives
	c. A mediated clinical eye
	d. Physical proximity
**3. Technologized, intimate patient-professional relationships**	a. Teleconsultation enables long-term engagement resulting in trustful relationships
	b. Feelings of intimacy
	c. Feelings of relief

### Transcending the institutional walls of home and hospital

The research material revealed the *transcendence* of institutional walls as a quality of teleconsultation in palliative homecare. Three subthemes could be discerned: the surplus value of teleconsultation technology (TCT) in enabling specialist care at home, the TCT’s incentive to join in condensed digital encounters, and the potential of TCT to jeopardize privacy.

Palliative patients and their caregivers valued the capacity of TCT to transmit specialist care to their homes, thereby obviating distressing hospital visits. Home is “the place where [they were] not dying men” (PA13), where the patients felt comfortable and in control.

Informal caregiver 3: “… at that moment, she was no longer capable of going to the hospital. And contact with the SPCT, through the video screen, came in handy then. Because if she would have to go to the hospital every time, she was just too tired for that.”

With regard to the TCT’s incentive to join in condensed digital encounters, participants emphasized that being able to see one another’s eyes, facial expressions, and situational contexts allowed them to be absorbed in an exclusive digital connectedness. Respondents deemed teleconsultation’s focused attention necessary to expose discrepancies in patients’ stories or potential dissimulations.

Patient 3: “You want to put on a brave front, and with the telephone, you’re always able to deviate from the truth. With this thing [TCT], they immediately see everything. … It is impossible to keep up appearances anymore, and somehow that is quite comforting. That they know immediately that you’re feeling miserable.”

Furthermore, the interviews and observations showed that TCT could jeopardize patient privacy. TCT opened up two social spaces, the home and hospital, which included, among others, co-present medical personnel and/or proxies, together with their prominent personal communication technologies (e.g., phones, pagers). Notwithstanding the hospital customs of asking consent for the presence of others and using a teleconsultation room, patients were sometimes surprised by unannounced intrusions of other people during teleconsultations, which resulted in privacy infringement. Informal caregivers played a significant role in creating a private conversation at home: some of them left the room to not overhear the teleconsultation between the patient and professional. Others accepted incoming calls at inconvenient moments, thereby exposing embarrassing situations.

Patient 1: “Yes, I think that’s just appropriate, [announcing] that there are more people overhearing [the teleconsultation]. … That’s privacy. It gets secured this way. You know about it. If you don’t want it, you can always say that.”

### Transparency of technology defines quality of care

With regard to TCT’s quality of transparency, the research revealed four aspects: the requirement of instant use; teleconsultation’s fit into the patients’ domestic lives; a mediated clinical eye; and physical proximity.

Readiness for instant use was occasionally compromised by hardware and software breakdowns, but transparency was most limited by a lack of Internet bandwidth: this low bandwidth created indefinable images and caused the sound to “break up into little pieces” (PA16). Such incomplete communication hampered “the medium [to] fade away, and [the conversation partner to] become increasingly visible” (PA13).

A large and immobile desktop device limited the capacity of teleconsultation to fit into the lives of weakened, often bedridden patients. In contrast to the tablet computers used later in the project, the immobile desktop device reminded some patients of their approaching death.

Patient 7: “[a teleconsultation device] is not something you have at home when you’re in full glory… It reminds you of going toward an ending.”

The analysis indicated that SPCT clinicians considered TCT images, which were limited to a patient’s head and upper chest, useful adjuncts for diagnosis. However, essential colors for diagnosis (e.g., yellow related to an icterus), small emotional cues, and physical indicators of decline (e.g., the fit of dentures) were often not discernible in these images. Nevertheless, SPCT clinicians did notice general physical progress or regression over time (e.g., blushing or weight loss) and/or mental states, as indicated on the patients’ faces (e.g., anxiety or delirium). They could also use TCT to determine the patients’ personalities and social contexts (household; home relationships) and to tend to the proxies and provide them with necessary counseling, to the delight of informal caretakers. In exchange, informal caretakers frequently functioned as prompters for patients, helping patients and clinicians to complete anamneses.

SPCT clinician 4: “And the image, it’s additional and supportive. You see someone’s status […], which makes the picture complete.”

The final aspect, (a lack of) physical proximity, was revealed when SPCT clinicians explained that they avoided discussing sensitive, emotional topics with particularly vulnerable patients via TCT because they did not feel sufficiently close to be able to comfort these patients. In contrast, these same patients reported that a physically distant, unobtrusive professional listener provided exactly the freedom needed to pour out one’s heart, after which “normal” life could be resumed.

SPCT nurse 1: “[a sigh] that was intense [and cries]. You just notice that you’re far away […]. That man will deteriorate rapidly from now on […]. I would really prefer to sit next to him now. A bit more personal. However, that’s how it is.”

Patient 1: “I cried during the conversation. [I: Yes, and afterwards…] I’m calm again. Relieved too […] You just have the opportunity to pour your heart out.”

### Technologized, intimate patient-professional relationships

Our research revealed that teleconsultation enabled long-term engagement that resulted in trustful relationships and fostered feelings of intimacy and relief.

In general, participants valued the repetitive character of teleconsultations (each new appointment meant recognizing that patients “would still be there”; IC17). Building a joint medical *and* interpersonal history with the same group of health care professionals facilitated engagement (e.g., incorporating the digital professional into domestic hospitality rituals), trust and tailor-made care. The patients referred to two preconditions for this history building: while following patients in their own homes, professionals should a) genuinely pay attention and b) avoid becoming patronizing. If these preconditions were met, dynamic, often-humorous discussions about the patients’ and professionals’ roles during teleconsultations occurred.

Patient 7: “You build a relationship together [with Nurse 1], let’s be honest. It wouldn’t be good if it wasn’t so. So you meet each other regularly. You become a little familiar with each other and more and more familiar in due time.”

According to patients, teleconsultations helped to overcome extreme introverted focus and offered consolation. Weekly teleconsultations inspired patients to actively highlight their values of life and death while being surrounded by the calm environment of their own homes. Moreover, seeing that the SPCT also addressed the issues of informal caretakers instilled a sense of relief in patients who understood their loved ones’ suffering.

Patient 13: “The screen goes off and I feel revived.”

The patients’ experiences of intimacy were nurtured by expressions of empathy from SPCT clinicians: by considering a patient’s unique situation, verbally and non-verbally (e.g., bending forwards, frowning) responding to patients’ emotions, and choosing considered opinions over pity (Q36).

Patient 7: “Of course. [Nurse 1] and I have become well acquainted. That’s what you get [with teleconsultation]. […] I think that if you meet each other daily, you’ll become freer with each other. I don’t tell a passer-by what my life has been like.”

Knowing that an SPCT was available via teleconsultation created a sense of safety and relief. The repetitive character of teleconsultation allowed it to serve as a reference point for instant discussion of symptoms and treatments. However, the onset of dying ultimately dispelled the patients’ desire for active participation.

IC5a: “And [the SPCT] can notice it immediately if something changes. It will be seen directly, if PA5 changes one way or the other. Instead of having to go to the hospital.”

## Discussion and Conclusions

This study demonstrated that three key components can describe the impact of teleconsultation on the home-based palliative care patient-palliative care specialist relationship: TCT enables patients and professionals to transcend the institutional walls of the home and hospital, TCT’s transparency changes the quality of care, and technologized but intimate patient-palliative care specialist relationships are real and prevalent in teleconsultations.

### The fit of real-time teleconsultation into palliative homecare

Specialist palliative care teleconsultations support a comfortable last phase of life, as they enable patients to receive expert care at home (in agreement with Clark [18]). Teleconsultations require the focused attention of professionals, which ideally leads to the refinement of their listening skills using visual clues [9,16]. In contrast to modern-day people’s tendencies to mainly use communication technologies for non-synchronous connections [33], this study’s patients aimed for synchronous connections with experts to truly expose themselves and no longer suffer in silence.

This study demonstrated that tablet computers best fit the praxes of SPCT clinicians. The tablets easily expose patients and patients’ lifestyles and social contexts. If SPCT clinicians apply the correct hermeneutics (i.e., knowing the particularities of TCT’s translation of a real situation into an image) [34], tailor-made distance care becomes possible. Tablet computers, rather than desktop devices, also support patients’ peace of mind, as these computers merged into the home environment and, therefore, did not remind them of their evolving medical condition [9,13,17,35,36] if privacy intrusions were avoided.

SPCT clinicians occasionally had reservations about addressing painful truths and emotional topics in teleconsultation due to the absence of physical proximity [9]. Even though the literature suggests that technology results in detached, dehumanized relationships [13,15,19,20], our study demonstrated that patients felt that the distance created by the technology was valuable free space wherein they could pour out their hearts, define their own roles, and co-design their own care in more equal patient-professional relationships. Teleconsultations enabled regular, timely “conversations to connect” with (the same) professionals [15,37] in which medical and personal engagement were principal drivers.

### Practical implications

**Fig 2:**
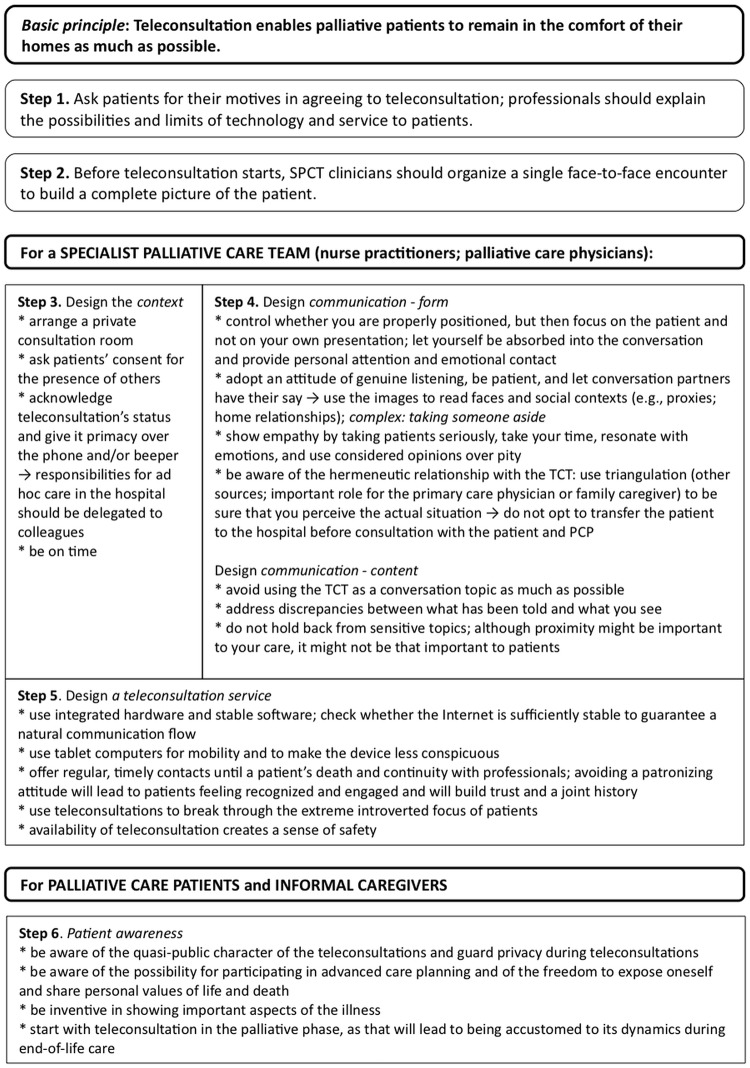
Practical implications: a step-by-step implementation guide for multidisciplinary and/or interdisciplinary palliative homecare by means of teleconsultation.

In the near future, patients will become more central to their own care via the use of modern communication technologies. Based on direct patient experiences, this study revealed the possibilities of teleconsultation provided that particular pitfalls are avoided. Both the pitfalls and the opportunities are summarized in an implementation guide to create tailor-made, multidisciplinary palliative care via the attentive use of teleconsultation (Fig 2).

### Study limitations

The design of the data collection was strictly planned, but real-life palliative care situations demanded a more flexible research approach that was focused on the well-being and increasing vulnerability of patients and their informal caregivers. Open follow-up interviews regularly replaced the semi-structured interviews. Although separate interviews with patients and informal caregivers were preferred during the study, dyad interviews (i.e., patients and informal caregivers) often occurred. This practice may have hampered the respondents’ freedom of expression, but dyad interviews also introduced meaningful additions, discussions, and insights into family dynamics [38].

Our sample may have contained selection bias because both the teleconsultation technology and the research generally attracted curious patients who were eager to control their care [18]. The change from a stand-alone desktop to a tablet computer largely improved patient acceptance and has lowered the threshold for participation during the study. The analysis showed sufficient diversity to secure theoretical profundity.

### Unanswered questions and future research

This paper focused on the relationship between SPCT members and home-based patients mediated by teleconsultation technology. The changes in relationships between the primary care physicians and hospital-based palliative care specialists due to teleconsultation will be discussed elsewhere (unpublished data).

This study about the fit of teleconsultation technology into palliative care introduced two new ethical questions. How does a teleconsultation technology, which naturally invites participants to active communication, relate to the emphasis on being *silent* with a patient as described in palliative care literature [20]? Furthermore, will technologized communication in palliative care contribute to a temporary reduction of suffering by providing patients with brief, precious reconnections with an understanding social environment, albeit a medical one [39]?

In conclusion, teleconsultation fits the practice of home-based palliative care. Teleconsultation, however, has to be carefully applied in line with this article’s implementation guide in order to succeed. Mediated by computer technology, teleconsultations enable focused attention and listening, empathic engagement between patient and palliative care specialists, and an opportunity for patients to co-design their own care within (the comfort of) their homes.

## Supporting Information

S1 Appendix The observation guide used for the long-term direct observations. (DOCX)

S2 Appendix The interview guide used for the semi-structured interviews and open follow-up interviews. (DOCX)
