# Supplementary material for: How Outpatient Palliative Care Teleconsultation Facilitates Empathic Patient-Professional Relationships: A Qualitative Study
Source: PLoS One. 2015 Apr 22;10(4):e0124387. doi: 10.1371/journal.pone.0124387 (PMC4406581; doi:10.1371/journal.pone.0124387)
Supplement: S1 Appendix — (DOCX) [file pone.0124387.s001.docx]

**Appendix 1**

**DIRECT OBSERVATIONS**

Based on Peterson MA. Anthropology & mass communication. Media and myth in the new millennium. 2003. New York: Berghahn Books.

| 1. Technology | a. specific technical constraints  b. their functions within modes of production  c. their signification as material objects.  d. their signification as communication modalities. |
| --- | --- |
| 2. Physical setting | a. integration of media technologies into social space (i.e. living room?, hospital?) |
| 3. Kinds of people | a. how are those involved organized?  b. how are those involved stratified? |
| 4. Acts | a. behaviors before/during/after teleconsultation  b. transitions from one activity to another (before/after teleconsultations) |
| 5. Patterns of interaction | a. within a social setting, but also through a medium.  b. (un)acceptable behavior |
| 6. Patterns of interpretation | a. articulation of teleconsultation technology assessments |
| 7. Vocabulary | a. the use of (verbal) symbols |
