# Supplementary material for: How Outpatient Palliative Care Teleconsultation Facilitates Empathic Patient-Professional Relationships: A Qualitative Study
Source: PLoS One. 2015 Apr 22;10(4):e0124387. doi: 10.1371/journal.pone.0124387 (PMC4406581; doi:10.1371/journal.pone.0124387)
Supplement: S2 Appendix — (DOCX) [file pone.0124387.s002.docx]

**Appendix 2**

**INTERVIEWS WITH PATIENTS AND SPCT MEMBERS**

The following guideline is used to initiate a first interview with all participants. As the research process and dying process continued, the interviewer chose specific topics from the initial interview/observations for further exploration. Respondents are invited to use everyday language and introduce topics which are important to them. In case of indistinctness, the interviewer uses probing questions, contrast questions and precise recapitulations to gain a deeper insight.

| Open questions | Issues that may be explored |
| --- | --- |
| Can you please tell me how the latest teleconsultation/video conversation went? | Discuss place and time (as a commodity); preparation for the conversation; what happened after the conversation; the value of a technical object in the room |
| Which persons were involved in the video conversation? | Taking initiative (dialogue); making health care choices |
| How do you relate to these people? | Recognition; commitment; engagement; professional attitude (empathy, et cetera); definition of good palliative care |
| Which topics did you discuss with each other? | Transparency about death and dying; establishing hope; making health care choices; ascertain awareness and understanding; transporting cultures |
| What did you see during the conversation? | People/places; hermeneutics (truthfulness of images); reducing parts of the world (what did you miss during the conversation?); transporting cultures |
| In what ways does a teleconsultation differ from a face-to-face encounter with a physician (at home or at the hospital)? |  |
|  | |
| Which people kept you company during the conversation?  Which role did they play in the conversation? | Relationship between patient and proxy; context of care |
| Additional questions for members of the palliative care team | |
| Could you describe the effect that you think teleconsultations have on your patients and/or their informal carers? | Transforming transmural care |
| How do teleconsultations influence your relationship with your patients and/or their informal carers? | ibid |
| How would you define contact/relationship with the primary care physicians during and beyond teleconsultations? | ibid |
| In what ways does teleconsultation contribute or hinder your daily care for your patients? | Ibid; could you please respond to the following proposition > teleconsultation contributes to further technologizing of palliative care |

**INTERVIEWS WITH PRIMARY CARE PHYSICIANS AND INFORMAL CAREGIVERS**

| Additional questions for primary care physicians | |
| --- | --- |
| Could you describe the effect that you think teleconsultations have on your patients and/or their informal carers? | Transforming transmural care |
| How do teleconsultations influence your relationship with your patients and/or their informal carers? | ibid |
| How would you define contact/relationship with the members of the specialist palliative care team during and beyond teleconsultations? | ibid |
| In what ways does teleconsultation contribute or hinder your daily care for your patients? | Ibid; could you please respond to the following proposition > teleconsultation contributes to further technologizing of palliative care |

| For informal carers: what roles did you play in the conversation? | |
| --- | --- |
|  | |
| Could you please show me ‘live’ what you do when preparing/using the teleconsultation technology? | A teleconsultation technology’s scripts |
| What possibilities/problems do you encounter while using the teleconsultation technology? | ibid |
| What other communication technologies do you have at your disposal? Could you compare these to using the teleconsultation technology? | Contrast question |
| When there’s room for retrospective questions: | |
| Till now, have you done anything you wanted to do with the teleconsultation technology? |  |
| Till now, were there any moments when you would rather be rid of the teleconsultation technology? |  |
| Till now, would you say the teleconsultations have been of value to you (or others around you)? | Relationship between teleconsultation and good palliative care |
